# Decomposing cross-country differences in quality adjusted life expectancy: the impact of value sets

**DOI:** 10.1186/1478-7954-9-17

**Published:** 2011-06-23

**Authors:** Richard Heijink, Pieter van Baal, Mark Oppe, Xander Koolman, Gert Westert

**Affiliations:** 1 Scientific centre for care and welfare (Tranzo), Tilburg University, Warandelaan 2, 5037 AB Tilburg, The Netherlands; 2 Centre for Prevention and Health Services Research, National Institute for Public Health and the Environment, PO Box 1, 3720 BA Bilthoven, The Netherlands; 3 Institute of Health Policy & Management and Institute for Medical Technology Assessment Erasmus University Rotterdam, PO Box 1738, 3000 DR Rotterdam, The Netherlands; 4 Expertise Centre for Methodology and Information Services, National Institute for Public Health and the Environment, PO Box 1, 3720 BA Bilthoven, The Netherlands; 5 Faculty Technology, Policy and Management, Delft University of Technology, Jaffalaan 5, 2628 BX Delft, The Netherlands; 6 Scientific Institute for Quality of Healthcare (IQ healthcare), Radboud University Nijmegen Medical Centre, PO Box 9101, 6500 HB Nijmegen, The Netherlands

## Abstract

**Background:**

The validity, reliability and cross-country comparability of summary measures of population health (SMPH) have been persistently debated. In this debate, the measurement and valuation of nonfatal health outcomes have been defined as key issues. Our goal was to quantify and decompose international differences in health expectancy based on health-related quality of life (HRQoL). We focused on the impact of value set choice on cross-country variation.

**Methods:**

We calculated Quality Adjusted Life Expectancy (QALE) at age 20 for 15 countries in which EQ-5D population surveys had been conducted. We applied the Sullivan approach to combine the EQ-5D based HRQoL data with life tables from the Human Mortality Database. Mean HRQoL by country-gender-age was estimated using a parametric model. We used nonparametric bootstrap techniques to compute confidence intervals. QALE was then compared across the six country-specific time trade-off value sets that were available. Finally, three counterfactual estimates were generated in order to assess the contribution of mortality, health states and health-state values to cross-country differences in QALE.

**Results:**

QALE at age 20 ranged from 33 years in Armenia to almost 61 years in Japan, using the UK value set. The value sets of the other five countries generated different estimates, up to seven years higher. The relative impact of choosing a different value set differed across country-gender strata between 2% and 20%. In 50% of the country-gender strata the ranking changed by two or more positions across value sets. The decomposition demonstrated a varying impact of health states, health-state values, and mortality on QALE differences across countries.

**Conclusions:**

The choice of the value set in SMPH may seriously affect cross-country comparisons of health expectancy, even across populations of similar levels of wealth and education. In our opinion, it is essential to get more insight into the drivers of differences in health-state values across populations. This will enhance the usefulness of health-expectancy measures.

## Background

Summary measures of population health (SMPH) have been calculated to represent the health of a particular population in a single number, combining information on fatal and nonfatal health outcomes [1,2]. SMPH have been applied to various purposes, e.g., to monitor changes in population health over time, to compare population health across countries, to investigate health inequalities (the distribution of health within a population), and to quantify the benefits of health interventions in cost effectiveness analyses [3-5]. In this study, we focus on using SMPH to compare the level of health across populations.

Although different types of SMPH have been developed [6-10], they usually comprise three elements: information on mortality, nonfatal health outcomes, and health-state values. Health-state values reflect the impact of nonfatal health outcomes on a cardinal scale, commonly comprising a value of 1 for full health and a value of 0 for a state equivalent to death. In SMPH, the number of years lived in a particular population (taken from life tables) is combined with information on the (proportional) prevalence of health states or diseases and the value of these nonfatal health outcomes. In this way, the number of life years lived in a population is transformed into the number of *healthy *life years lived.^1 ^The value sets provide the link between the information on nonfatal health outcomes and the information on mortality.

There has been much debate on SMPH, in particular regarding the validity, reliability, and cross-country comparability of different methods. A complete discussion on the pros and cons of different methods is beyond the scope of this paper and can be found elsewhere [6,11,12]. In short, crucial and persistent issues have been the measurement and valuation of nonfatal health outcomes and the incorporation of other values such as discounting or equity. In cases where SMPH are used to compare population health across countries, it is essential to use the same concepts and measurement methods for mortality, nonfatal health outcomes, and value sets across countries. Furthermore, it is crucial to understand in what way the method chosen may affect cross-country variation in the summary measure.

In this study we performed a cross-country comparison of Quality Adjusted Life Expectancy (QALE). We included information on health-related quality of life (HRQoL) to represent nonfatal health outcomes. EQ-5D (HRQoL) population surveys were used, and we included the 15 countries in which an EQ-5D population survey had been conducted. The EQ-5D is a standardized and validated questionnaire for measuring HRQoL. It comprises five dimensions such as mobility and self-care. The information on HRQoL, in combination with one of the available value sets, can be used to calculate QALE. As far as we know, a HRQoL-based approach has rarely been used in SMPH [1], particularly in international comparisons. The approach may prove interesting, since the value sets are calculated on the basis of choice-based methods, which have a theoretical foundation in economic theory [13]. Furthermore, data requirements of an EQ-5D type of instrument may be limited compared to other approaches such as using disease prevalence, particularly in international comparisons [14,15]. There are several other validated HRQoL instruments besides the EQ-5D, such as the SF-36 and the Health Utility Index mark 2 and mark 3 (HUI-2 and HUI-3) [16-18]. Muennig et al. used EQ-5D data to estimate Health Adjusted Life Years (HALY) in the American population [19]. They found differences across income groups, yet they did not provide insight into the uncertainty in their estimates. In Canada, the HUI was used to calculate a national SMPH [20,21]. Feeny et al. used the HUI-3 and a single Canadian value set to compare health expectancy between Canada and the US [21]. Significant health differences between the two countries were found. Health-state profiles have also been included in SMPH in combination with information on diseases and disability [7].

Our first aim was to provide more empirical evidence on international differences in HRQoL-based health expectancy. Additionally, we aimed to explore the impact of the value set choice. In the context of international comparisons, a choice has to be made between country-specific values and cross-country (global) values. The issue of value set choice has not been extensively discussed in the literature, however. It can be argued that if SMPH serve (international) health system performance assessments, country-specific value sets are preferred. Health systems should deliver outcomes in accordance with the preferences of the population they serve and whose means are put in use. Country-specific value sets may not always be available, however. Some have used foreign value sets, e.g., from neighboring countries. For example, Feeny et al. compared health-utility-based health expectancy between the US and Canada using the Canadian value set for both countries [21]. The authors remarked this as a limitation because the true preferences of the US population may not exactly resemble the Canadian values. Some have used a single global value set in international comparisons. For example, Mathers et al. calculated Health Adjusted Life Expectancy (HALE) by combining data on disease incidence (from the WHO Global Burden of Disease [GBD] study) with, for a subset of countries, survey data on health states [7]. Global value sets were applied to both the diseases (values were called severity weights in this context) and the health states. International comparisons of disability-adjusted life years (DALYs) and of disability-adjusted life expectancy (DALE) also used a single value set across countries [22-24]. It has been argued that the valuation of health domains shows reasonable consistency across countries, justifying the use of a global value set from an empirical perspective [25]. Nevertheless the need for more empirical evidence was acknowledged. Others did find differences in disease/disability-related values across countries and raised doubts about the universality of health values [26]. Another consideration that could support the use of global values is that identical interventions on identical patients will result in different benefits if different value sets are used. For example, less-healthy (poorer) populations may experience a smaller impact of health problems and a smaller benefit from interventions because they are unaware of better health outcomes. In other words, differences in values and expectations would determine system performance and could also alter resource allocation decisions across populations in a way that may be considered undesirable.

In summary, the literature has demonstrated a need to improve the understanding of differences in the valuation of health, also in the context of international comparisons of SMPH [25-27]. We aimed to provide more empirical evidence on the impact of value sets on cross-country differences in health expectancy. Furthermore, we aimed to discuss these results in the context of the theoretical and methodological issues that have been raised in the literature.

## Methods

### Data

We calculated QALE in 15 countries using individual-level EQ-5D survey data (provided by Euroqol Group) and life tables from the Human Mortality Database (HMD) [28]. The HMD did not provide life tables for Armenia and Greece, for which we instead used WHO life tables [29]. The countries were selected on the basis of EQ-5D data availability. The EQ-5D surveys were conducted between 1993 and 2002 (see Additional file 1). All surveys used the standard EQ-5D setup. The translation process of the EQ-5D surveys followed the guidelines proposed in the international literature [30]. Survey respondents were noninstitutionalized persons older than 18 years. Sample size varied between 400 and 10,000 observations per country (see Additional file 1). We excluded 2,989 observations with missing values in at least one of the EQ-5D dimensions because HRQoL could not be calculated in these cases. Consequently 41,562 observations/individuals remained in the pooled dataset. We used life tables from the year 2000 for all countries.

The value sets used to weight health states were all based on the time trade-off (TTO) elicitation technique and were taken from the literature. TTO-based valuation studies had been conducted in Germany, Japan, the Netherlands, Spain, the UK, and the US (see Table 1) [16,31-35]. The TTO method is considered the most appropriate (consistent) method to elicit preferences, compared to the Standard Gamble technique or the Visual Analogue Scale, for example [36].

**Table 1:** Characteristics of the TTO value sets

Country	Reference	Elicitation year	Minimum HRQoL
Germany	Greiner (2005)	1997-1998	-0.205
Japan	Tsuchiya (2002)	1998	-0.111
Netherlands	Lamers (2005)	2003	-0.329
Spain	Badia (2001)	1996	-0.654
UK	Dolan (1997)	1993	-0.594
US	Shaw (2005)	2002	-0.102

### HRQoL

The EQ-5D comprises five domains: mobility, self-care, usual activities, pain/discomfort, and anxiety/depression. Each domain contains three levels: no problems (1), some problems (2), and extreme problems (3). For example, a respondent may report no problems in mobility, self-care, usual activities, and pain/discomfort, and some problems in anxiety/depression. Generally the five answers are transformed into a single HRQoL index as follows:(1)

where *α_cjk _*= value of EQ-5D domain *j *and level *k *for country *c*; *d_jk _*= dummy for health state *j *and level *k*; *β_c _*= value of having some or severe problems in at least one health domain (dummy *N2*) for country *c*; and *γ_c _*= value of having severe problems in at least one health domain (dummy *N3*) for country *c*.

The US value set was based on a different formula [35]:(2)

where *D*1 = number of domains with some or extreme problems beyond the first, *I2square *equals the square of the number of domains at level 2 beyond the first, and *I3square *equals the square of the number of domains at level 3 beyond the first. This model was chosen in the US because it provided the best fit for the data [35]. Additionally, in contrast to the other value sets, the US model was meant to take account of the marginal changes in HRQoL associated with having some or extreme problems in additional domains.

**Figure 1:**
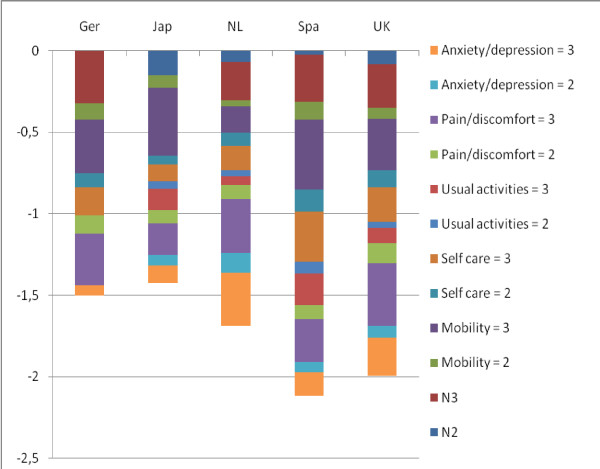
***Value of the EQ-5D domains and levels***. ^1^The US values are not shown because they are based on a different formula.

Equation (1) and equation (2) show that the maximum HRQoL equals 1. The values *α_cjk _*reflect the HRQoL reduction associated with having some problems or severe problems in each EQ-5D domain. These preferences may differ across countries as shown in Table 1 by the difference in minimum HRQoL (see also [34,37,38]). Figure 1 demonstrates the relative value of each EQ-5D dimension for the five value sets that are based on equation (1). For example, it shows that, compared to Dutch residents, people in the UK attached greater value to having some or severe health problems in all domains except anxiety (see [33]). Consequently, minimum HRQoL was lower in the UK (-0.594 vs. -0.329).

### Analysis

We used the Sullivan approach to combine mortality and nonfatal health outcomes and to calculate QALE [39]. The life tables comprised current death rates and conditional probabilities of death by country, gender, and age group (mostly five-year age groups). These probabilities were used to calculate the number of life years lived per age group for a hypothetical cohort. We multiplied the number of life years, as given in the HMD life tables, with the mean HRQoL as predicted by the parametric model described underneath, in order to calculate the number of healthy life years. Finally, the total number of healthy life years *from *age × was divided by the number of survivors in the hypothetical cohort at age × to calculate QALE at age x. We excluded age groups under 20 years, because the EQ-5D surveys were conducted among individuals older than 18 years. In addition, we were unable to differentiate HRQoL in the age groups over 85 years, because the maximum age of respondents was 90 in almost all surveys. Equation (3) is a formal representation of the QALE.(3)

*LY_c,g,a _*equals total number of life years lived in country c, gender g, and age group a;

*HRQoL_c,g,a _*equals average (predicted) HRQoL by country c, gender g, and age group a;

*l_c,g,a _*equals number of survivors in the life table cohort for country c, gender g, and age group a; and *z *equals the last open-ended age interval of the life table.

*HRQoL_c,g,a _*was calculated in three steps: 1) we calculated HRQoL at the individual level using equation (1); 2) we estimated the predicted HRQoL at the individual level using a multiple regression model; and 3) we computed the mean predicted HRQoL by country, gender, and age. In step 2, we estimated a multiple regression model with HRQoL as dependent variable (in the range [minimum, 1]) and age, gender, country dummies, and education level as independent variables. We estimated the model to fully exploit the information available in the pooled dataset and to explore the relationship between HRQoL and respondent characteristics (Additional file 2 shows that there is almost no difference between QALE using observed HRQoL and QALE using predicted HRQoL). Previous studies have shown that HRQoL is associated with demographic and socioeconomic characteristics such as age, gender, education, income, and race (e.g., [19,40-42]). The EQ-5D surveys provided information on the respondents' age (the average age was 47 in the pooled dataset), gender (46% male), country, and level of education (primary education 31%, secondary education 57%, and university level 12%). The variables socioeconomic status and smoking status were not used because of high nonresponse rates (43% and 47% respectively). It was expected that the relationship between HRQoL and, for example, age differed by gender and country. Therefore interaction terms between country, gender, and age were included in the model. We used nonparametric bootstrap techniques to calculate 95% confidence intervals. As discussed in Pullenayegum et al., regression models that use this type of outcome measure need to take heteroscedasticity and a nonnormal distribution into account [43]. Pullenayegum et al. showed that OLS regression with nonparametric bootstrap can give 'acceptable adequacy' of the confidence intervals with these data. We also tested alternative models, a tobit model and a two-part model, which have been used to model skewed and truncated data. The outcomes of these models did not alter the main results and conclusions (these regression results can be obtained through the corresponding author).

Finally, we computed counterfactual estimates in order to explore the contribution of mortality, health states, and health-state valuation to cross-country variation in QALE. In this part of the study, we only included the six countries for which value sets had been established (Table 1). As a result, six sets of counterfactual estimates were generated. In each set, a different country was used as reference country. Suppose we use Germany as reference country. Then, we imputed mortality rates, health-state profiles, and values from Germany into QALE of, for example, Spain. Subsequently, we investigated the associated change in QALE for Spain in comparison to QALE based on Spanish mortality, health states, and values.

In the first counterfactual estimate we used country-specific value sets, country-specific EQ-5D health states, and death rates of the reference country. In other words, we imputed *LY *and *l *of the reference country in equation (3). The difference between this counterfactual QALE and the original QALE (based on country-specific mortality, health states, and values) revealed the contribution of mortality. With the second counterfactual QALE we estimated the impact of health states using country-specific value sets, country-specific death rates, and EQ-5D health states of the reference country. Now the HRQoL component in equation (3) was based on country-specific values *α_c,j,k _*and on the health state profiles *d_jk _*of the reference country. The difference between this counterfactual QALE and the original QALE showed the contribution of health states. The third counterfactual estimate comprised country-specific EQ-5D health states, country-specific death rates, and the value set of the reference country. We imputed the values *α *of the reference country in equation (1). Subsequently, QALE was estimated using equation (3) and the difference between this counterfactual QALE and the original QALE demonstrated the impact of value sets.

## Results

### Regression results

Table 2 presents the results of the regression model (using UK values). The table shows that HRQoL declined with age, although the relationship was not linear (age, age squared, and age cubic were jointly significant). The gender-age interaction term shows that the age effect differed between men and women: the reduction in HRQoL over age was somewhat smaller for males. In addition, the regression results showed significant country effects and cross-country differences in the impact of age and gender. The country dummies and interaction terms were jointly significant. HRQoL was also positively associated with education level.

**Table 2:** Regression results^1^

Main effects	Coef.	*P > |z|*	Interaction terms	Coef.	*P > |z|*
Age	-0.069	0.000	Gender*age	-0.003	0.000
Age squared	0.003	0.004			
Age cubic	-0.000	0.002	Belgium*age	0.028	0.000
			Canada*age	0.027	0.000
Education^2^	0.040	0.000	Finland*age	0.024	0.000
Gender^3^	0.010	0.555	Germany*age	0.026	0.000
			Greece*age	0.020	0.000
Belgium	-0.114	0.003	Hungary*age	0.018	0.000
Canada	-0.107	0.000	Japan*age	0.032	0.000
Finland	-0.078	0.010	Netherlands*age	0.031	0.000
Germany	-0.086	0.009	New Zealand*age	0.027	0.000
Greece	0.018	0.700	Slovenia*age	0.020	0.000
Hungary	-0.025	0.372	Spain*age	0.029	0.000
Japan	-0.085	0.042	Sweden*age	0.033	0.000
Netherlands	-0.125	0.000	UK*age	0.026	0.000
New Zealand	-0.104	0.003	US*age	0.025	0.000
Slovenia	-0.114	0.003			
Spain	-0.090	0.001	Belgium*gender	-0.001	0.966
Sweden	-0.189	0.000	Canada*gender	-0.015	0.490
UK	-0.094	0.001	Finland*gender	0.008	0.689
US	-0.132	0.000	Germany*gender	-0.008	0.724
			Greece*gender	-0.017	0.496
			Hungary*gender	-0.024	0.160
			Japan*gender	-0.009	0.701
			Netherlands*gender	-0.015	0.397
			New Zealand*gender	0.015	0.502
			Slovenia*gender	0.019	0.367
			Spain*gender	-0.024	0.158
			Sweden*gender	0.036	0.037
			UK*gender	0.023	0.215
			US*gender	-0.014	0.447
Constant	1,138				
Adj R-squared	0.16				
N	40,650				

¹ Standard errors were calculated using non-parametric bootstrap technique

^2 ^Education levels: 1 = low (primary); 2 = medium (secondary); 3 = high (university)

^3 ^Gender: 0 = male; 1 = female

### QALE

**Figure 2:**
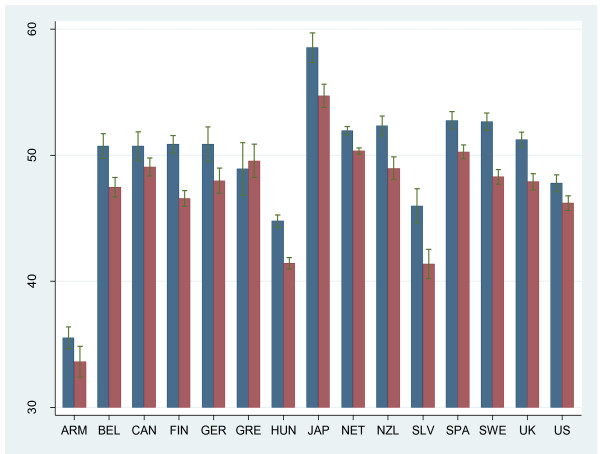
***Quality Adjusted Life Expectancy at 20 years by country and gender*^*1*^**. ^1^Confidence interval based on nonparametric bootstrap technique. Blue: females, Red: males.

Figure 2 shows QALE at age 20 by country and gender (using UK values). It shows that QALE at age 20 ranged from 33 years in Armenia (males) to almost 61 years in Japan (females). The figure shows that QALE at age 20 years was higher for females than for males. Only Greece showed a higher male QALE, yet the confidence intervals of the two genders largely overlapped for this country. The absolute gender difference in QALE ranged between 1.6 years in the US and 4.6 years in Slovenia.

### Value set choice

The former results were calculated using the UK value set in all countries. Table 3 demonstrates QALE using different value sets. The table shows that the UK value set generated the lowest QALE in most (67%) of the country-gender strata. The German value set generated the highest QALE in all country-gender strata, with a maximum difference of 7.2 healthy years (difference in QALE between the German value set and the UK value set for females in Armenia). The US value set consistently showed the second-highest QALE. In 60% to 70% of all country-gender strata, the Spanish value set ranked third, the Dutch value set ranked fourth, the Japanese value set ranked fifth, and the UK value set ranked sixth. The relative change in QALE, as a result of a change in value set choice, varied between countries. For example, the difference in QALE between the German value set and the UK value set was close to 3% for Japanese males, but more than 20% for Armenian females. We also added a country ranking (R) by value set and by gender. The countries at the top end and low end of the ranking showed a stable position across value sets. In between, the ranking of the countries was affected to some extent. Around 50% of the country-gender strata moved two or more rank-positions across value sets. Notable rank-changes were found for Belgium (females), Canada (females), Finland (females), Greece (males), and Sweden (males).

**Table 3:** QALE at age 20 years using different value sets plus a country ranking (R)^1^

	Value set Germany		Value set Japan		Value set Netherlands		Value set Spain		Value set UK		Value set US	
	QALE	R	QALE	R	QALE	R	QALE	R	QALE	R	QALE	R
*Males*												
ARM	39.13	15	36.93	15	34.91	15	35.99	15	33.62	15	37.85	15
BEL	50.88	9	47.22	10	48.45	10	49.19	10	47.47	10	49.23	10
CAN	52.72	2	49.00	5	49.89	6	50.76	5	49.07	5	51.02	4
FIN	49.71	11	46.35	12	48.00	11	47.97	11	46.57	11	48.47	11
GER	**50.68**	**10**	48.21	9	49.24	7	49.51	8	47.98	8	49.83	9
GRE	51.20	7	50.17	4	49.95	5	49.72	7	49.54	4	50.81	5
HUN	44.34	14	41.83	13	42.07	14	42.60	14	41.42	13	43.12	14
JAP	56.14	1	**54.68**	**1**	55.19	1	55.43	1	54.70	1	55.46	1
NET	52.60	5	50.25	3	**51.33**	**2**	51.52	3	50.34	2	51.66	2
NZL	52.27	6	48.82	6	50.13	4	50.45	6	48.96	6	50.74	6
SLV	46.04	13	41.36	14	42.74	13	42.73	13	41.37	14	43.96	13
SPA	52.66	3	50.43	2	51.17	3	**51.57**	**2**	50.27	3	51.65	3
SWE	52.63	4	48.37	8	49.11	8	50.84	4	48.29	7	50.48	7
UK	50.93	8	48.60	7	48.95	9	49.22	9	**47.89**	**9**	49.94	8
US	49.67	12	46.61	11	47.33	12	47.90	12	46.20	12	**48.39**	**12**
*Females*												
ARM	42.74	15	39.43	15	37.03	15	38.87	15	35.51	15	40.96	15
BEL	55.14	7	50.77	10	52.24	8	53.08	6	50.73	10	53.17	10
CAN	55.50	5	50.83	9	52.05	10	52.96	9	50.73	9	53.51	7
FIN	54.70	10	50.95	8	52.69	6	52.49	10	50.87	8	53.36	8
GER	**55.12**	**8**	51.22	7	52.12	9	53.06	7	50.88	7	53.35	9
GRE	51.41	13	49.98	11	50.23	11	50.23	11	48.91	11	50.80	12
HUN	49.69	14	46.01	14	45.65	14	46.89	14	44.78	14	47.87	14
JAP	61.01	1	**58.68**	**1**	59.53	1	59.87	1	58.54	1	59.99	1
NET	55.35	6	52.10	4	**53.44**	**5**	53.59	5	51.94	5	54.11	5
NZL	56.45	4	51.99	5	53.55	4	54.11	4	52.32	4	54.51	4
SLV	51.88	12	46.03	13	47.64	13	47.60	13	45.99	13	49.22	13
SPA	56.67	3	53.80	2	53.93	2	**54.80**	**3**	52.76	2	55.32	2
SWE	56.75	2	52.97	3	53.70	3	55.04	2	52.67	3	54.93	3
UK	54.98	9	51.75	6	52.27	7	52.98	8	**51.23**	**6**	53.56	6
US	52.45	11	48.92	12	49.18	12	50.03	12	47.79	12	**50.93**	**11**

^1 ^QALE in bold where country-specific values were used.

### QALE decomposition

**Figure 3:**
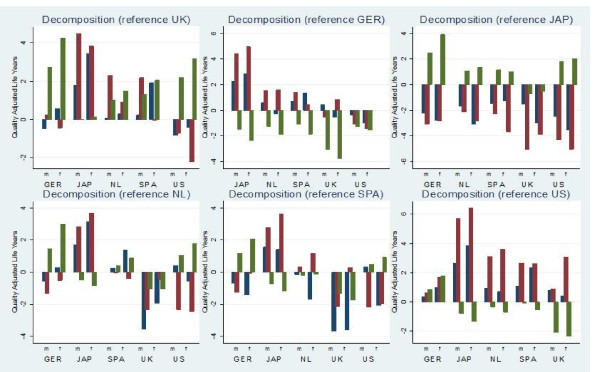
***Contribution of mortality, EQ-5D health states and value sets to cross-country differences in QALE^1^***. ^1^The y-axis shows the difference in quality adjusted life years between the QALE that comprised country-specific components and each counterfactual estimate. Blue: mortality, Red: health states, Green: values.

Counterfactual estimates were generated in order to explore the role of mortality, health states, and health-state values in cross-country differences. Figure 3 demonstrates the results. Each of the six countries involved (Germany, Japan, Netherlands, Spain, UK, and US) appears once as reference country in the counterfactual scenarios. As a result, six figures are shown. The figure demonstrates that the impact of the different QALE components varied substantially across countries. For example, the top-left graph demonstrates the contribution of mortality, EQ-5D health states, and health-state values to the difference in QALE with the UK. It shows that mortality rates explained the major part of the QALE difference with the UK for Japanese females and Spanish females. Differences in terms of valuation explained most of the difference in QALE with the UK for Germany and the US. Differences in EQ-5D health states explained the greater part of the variation in QALE for males in Japan, the Netherlands, and Spain. The figure shows that the differences in QALE with Germany are largely explained by the valuation component for all countries.

## Discussion and conclusions

In this study we performed an international comparison of HRQoL-based health expectancy. We found that QALE at age 20 ranged between 33 years in Armenia and almost 61 years in Japan. Generally, female QALE was higher than male QALE within this set of countries. In terms of QALE, Hungary and Slovenia performed better than Armenia, yet worse in comparison to the other countries. The relatively low health expectancy for a country such as Armenia may be expected given its lower levels of health spending and national income and its different socioeconomic circumstances. The United States performed worse in terms of QALE compared to the other western high-income countries in the dataset. Many studies have found such unfavorable health outcomes in the US and several explanations for this phenomenon have been given, such as an inefficient health care system, substantial disparities in the population in terms of access to health care, or behavioral factors (unhealthy diets) [44,45].

In the final part of the analysis, we decomposed the difference in QALE using counterfactual scenarios. It was shown that the relative contribution of mortality, health states, and health-state values differed among countries. For example, the high QALE for Japanese males was to a large extent a result of a low prevalence of health problems in EQ-5D domains. In turn, the better average health of Spanish females was largely explained by lower mortality rates. Interestingly, in various cases the EQ-5D profiles showed a greater contribution to differences in QALE than differences in mortality. Lower mortality did go hand in hand with better HRQoL, although there were exceptions. For example, Dutch females had a lower life expectancy than Spanish females, yet they experienced fewer health problems in EQ-5D domains. As a result, the difference in HRQoL-based health expectancy was smaller than the difference in life expectancy between these two countries. The decomposition confirmed that international comparisons of health expectancy, based on country-specific values, are influenced substantially by differences in value sets.

Differences in health expectancy across countries may stem from various factors, among which methodological issues and cultural differences play a role. Amid the three main SMPH elements (mortality, nonfatal health outcomes, and valuation) we focus on the value sets first. A remarkable result was the difference in QALE across the six TTO value sets. The German value set generated QALE up to seven years higher than the UK value set. The ranking of countries varied to a lesser extent across value sets, particularly in the high-performing or low-performing countries. We did find rank switches in the group of average performers. This may be expected because the differences in QALE were relatively small in this middle group, showing various overlapping confidence intervals (see Figure 2). Therefore, the ranking of these country-gender strata is particularly sensitive to the value-set choice. Around 50% of the country-gender strata showed a rank-change of two or more positions across value sets. Interestingly, the relative change in QALE associated with the value set choice differed across countries. The impact was greatest in low-performing countries such as Armenia, Hungary, and Slovenia. We also found that the ranking of countries did not consistently improve when local values were used. For example, Germany did not reach a higher rank in the German value set compared to the ranking in which Japanese values were used.

In the literature, the variation in health valuation has largely been explained by methodological differences across valuation studies and differences in the level of wealth and the level of education among populations [27]. In our case the available value sets represented the preferences of Western countries of similar levels of education and similar levels of wealth. Although we cannot exclude that methodological differences played a role, we argue that these cannot fully explain the variation that was found (see also [46]). All studies were conducted using face-to-face interviews, applied the TTO technique to elicit values, and included nationally-representative samples. In order to determine the valuation function, they used similarly specified least squares regression models representing the relationship between the TTO outcome and EQ-5D domains-levels and took account of within-individual error correlation [46]. The main difference was the model used in the US, which included a different specification of the N2 and N3 interaction terms and the marginal HRQoL effects. The US value set took account of a decrease in the marginal reduction in HRQoL associated with further increases in the number of domains with any problems or extreme problems. Still, the extent to which the US valuation function generated different HRQoL scores not only depended on the interaction terms and marginal effects, but also on the values attached to the individual domains and levels. Additional file 3 shows for each value set the HRQoL score associated with certain health states to exemplify the differences.

Consequently, we argue that a more conceptual discussion is needed. Cross-country variation in values may reflect cultural differences or differences in the availability of certain social services (and therefore the perceived/expected impact of health impairments). Naturally, health-state values also differ among individuals [47]. It may be argued that national or global value sets should cover this within-population variation in terms of values. In other words, the samples in elicitation studies need to be representative along the relevant population characteristics (similar to the other elements of SMPH). The cross-national differences in values need to be taken into account in the context of health-system-performance assessments and international comparisons of population health. In such studies, country-specific value sets may be preferred, since each health system should deliver outcomes according to the preferences of the population it serves and whose means are put in use. Moreover, the varying impact of health problems across countries needs to be accounted for. Some previous international comparisons of SMPH have used global value sets, based on the argument that health values are reasonably consistent across countries. However, the result of this study, similar to, for example, Üstün et al. [26], points to the contrary and shows that variation in values may affect SMPH outcomes. A drawback of using country-specific value sets is that they may not always be available, as was experienced in this study and in previous studies (e.g. [21]). In our opinion, the best solution is to calculate health expectancy by different foreign value sets and to compare the differences (as in Table 3). Additionally, the use of country-specific value sets in international comparisons may deserve close scrutiny from an equity perspective, particularly if there is a relationship among values, true health status, and level of wealth. Populations with less exposure to what constitutes "full health" may assign lower values, i.e., a smaller loss in terms of HRQoL, to certain health problems. As a result, a particular health intervention will generate fewer benefits in these populations. From an equity perspective, this may be considered undesirable. This argument has not been tested empirically though, and may be less relevant when only high-income countries of similar levels of health are included, as in our study.

The issue of value-set choice not only pertains to HRQoL-based health expectancy. All SMPH using multiple health states, diseases, levels of disability, or other morbidity measures use a valuation function or a set of weights. Only measures such as disability-free life expectancy do not comprise value sets. Such approaches classify people in two groups: with or without disability or disease. In that case you simply multiply the proportion without any disability with the number of life years lived in a particular stratum. Obviously these are rather crude methods that neglect differences in severity levels.

Two other issues need to be raised regarding the valuation part of SMPH. First, a plus of the EQ-5D type instrument, particularly in case an economic perspective is required, may be that value sets have been elicited using a choice-based method (TTO technique). Choice-based methods are considered the preferred method among economists to elicit people's preferences. The extent to which the elicitation method affects cross-country differences is largely unknown. Some have argued that different elicitation methods generate a rather similar cross-country variation in terms of values, but more research is needed on this issue [47]. Secondly, we need to address the question of whose values should be used. The value sets we used all represented general population values. Various authors have compared population values with patient values [48-51]. From an economic perspective, population values may be preferred, since health systems consume public means and should therefore allocate their resources and outcomes according to the preferences of the general population [48]. However, it was found that the general public attaches a much greater loss in terms of HRQoL to particular health problems than patients do. Although patients are better informed about the impact of morbidity, the adaptation effect is present among them [52,53]. Expert opinion has also been applied in previous international studies on SMPH [24]. The question is to what extent experts are able to assess the impact of different health states or diseases on people in general as well as for different populations. As a result this discussion appears unresolved.

As demonstrated by the decomposition, differences in QALE are also affected by differences in health states. Two major measurement issues should be discussed in this respect. First, although all studies used the same standardized EQ-5D instrument, the mode of administration differed across studies. It has been shown that telephone surveys in particular may generate more positive HRQoL scores compared to self- or interviewer-administered surveys [54]. The surveys included in our study were conducted as face-to-face interviews (Armenia, Greece, Japan, Spain, and UK) or self-administered postal interviews (other countries). Only part of the German data was based on a telephone survey. A second major measurement issue regarding the measurement of nonfatal health outcomes is response heterogeneity. People who are in an objectively equal health state may respond differently to the same health question. Response heterogeneity can be explained by differences in norms and expectations, in awareness, and in access to health care across populations. It may affect the validity and the cross-population comparability of all SMPH using self-reported health data (in terms of health states, disability, or disease) [55]. At the same time, the effect of response heterogeneity may somewhat be dampened if similar mechanisms also play a role in the valuation of nonfatal health outcomes. Some have argued that response heterogeneity may be less of a problem if different severity levels are included in the morbidity measure, since most threshold issues arise at the lower-valued mild-severity levels [1]. Moreover, the problem may be greater in self-rated general health questions, and some authors even used EQ-5D type of questions as more objective health measures [56,57]. Still, it remains unclear to what extent the reporting of EQ-5D health states, and our international comparison, have been subject to response bias. Whether response bias in the measurement of morbidity is related to the variation in the valuation of morbidity needs further investigation.

From a practical point of view, HRQoL-type of data may be preferred, since this approach may turn out to be less resource-intensive in terms of data gathering and data analysis than, for example, disease-based methods [22]. The latter approach requires information on many types of diseases and on the impact of all diseases in terms of disability. At an international level, data availability may be limited, which could cause less accuracy of the results. Furthermore, the presence of comorbidity complicates disease-based calculations [58]. In turn, an advantage of disease-based measures may be that clinical records or administrative records on the prevalence of diseases can be used. Such data do not suffer from self-report problems.

The following should be kept in mind while interpreting our results. First, the EQ-5D surveys were conducted in different years. This also holds for the value sets that were used, whereas preferences may change over time. It is unclear whether this is the case and to what extent this may have affected the results. We did see that value sets from similar years still showed substantial differences such as those from the Netherlands and the US or those from Germany and Japan. Future research could clarify to what extent health-related preferences change over time. Secondly, certain population groups were not included in the EQ-5D samples, such as inhabitants younger than 20 years and, in most surveys, people older than 85. Therefore we did not calculate QALE at birth and were unable to differentiate HRQoL within the 85-plus group. In addition, the surveys did not include the institutionalized population. However, due to a lack of comparable data, it is unclear to what extent this influenced the cross-country variation. Further, it was unclear whether all potential determinants of HRQoL were represented sufficiently. Thirdly, we did not take uncertainty in mortality into account because this information was not included in WHO life tables. However, there will be little uncertainty in life tables given the large population size. Consequently, the uncertainty in health expectancy particularly arises in the morbidity part of these measures [21]. Finally, as discussed before, different researchers may have used slightly different protocols and analyses which may have affected the differences in value sets [46].

In conclusion, we recommend that future international comparisons on SMPH profoundly discuss their value-set choice, including the theoretical and practical issues, and perform sensitivity analyses where possible and necessary. In addition, more qualitative research on the determinants of differences in valuation within and across populations is needed. This will improve the interpretation and the usefulness of HRQoL-based, and other, summary measures of population health.

## Competing interests

The authors declare that they have no competing interests.

## Authors' contributions

RH performed the analyses and wrote the draft manuscript. MO collected the data. All authors have made substantial intellectual contribution to the manuscript. All contributed to the conception and design of the study and the interpretation of the data. All authors have been actively involved in drafting or revising the manuscript and have given final approval of the version to be published.

## Endnote

^1^A simplified example: suppose that the life expectancy at birth of a population is equal to 80 years. Furthermore assume that half of the population lives in perfect health for 80 years, and the other half lives in an imperfect health state for 80 years. If the value of this imperfect health state is 0.5 then half of the population will live 80 healthy years and half of the population will live 80*0.5 = 40 healthy years. Consequently health expectancy of the entire population will be 60 years.

## Supplementary Material

Additional file 1 **Characteristics of the surveys included in the dataset**. The table shows in which year the EQ-5D surveys were conducted and it shows the sample size of the survey for each country. Click here for file

Additional file 2 **Observed and predicted HRQoL and QALE by country, gender and age (UK value set)**. The figures show the observed HRQoL by country, gender and age group. Additionally, the HRQoL by country, gender and age group as predicted by the regression model is shown (the line in each figure). The last figure demonstrates QALE at age 20 using the observed HRQoL vs. QALE at age 20 using the predicted HRQoL. Click here for file

Additional file 3 **HRQoL score associated with different EQ-5D profiles according to six value sets**. HRQoL score associated with different EQ-5D profiles according to the six value sets. Each point on the x-axis represents a hypothetical set of answers in the five EQ-5D domains: mobility, self-care, usual activities, pain/discomfort and anxiety/depression. Each domain contains 3 levels: no problems (1), some problems (2), and extreme problems (3). Click here for file
