# Supplementary material for: Decomposing cross-country differences in quality adjusted life expectancy: the impact of value sets
Source: Popul Health Metr. 2011 Jun 23;9:17. doi: 10.1186/1478-7954-9-17 (PMC3146826; doi:10.1186/1478-7954-9-17)
Supplement: Additional file 1 — Characteristics of the surveys included in the dataset. The table shows in which year the EQ-5D surveys were conducted and it shows the sample size of the survey for each country. [file 1478-7954-9-17-S1.DOC]

**Characteristics of the surveys included in the dataset**

| **Country** | **Year** | **Sample size** |
| --- | --- | --- |
| Armenia | 2002 | 2222 |
| Belgium | 2001 | 1241 |
| Canada | 1997 | 1472 |
| Finland | 1992 | 2325 |
| Germany | 1994-1998 | 800 |
| Greece | 1998 | 464 |
| Hungary | 2000 | 5202 |
| Japan | 1998 | 620 |
| Netherlands | 2001 | 9540 |
| New Zeeland | 1999 | 1328 |
| Slovenia | 2000 | 742 |
| Spain | 1996-2000 | 2732 |
| Sweden | 1994-1998 | 3497 |
| UK | 1993 | 3381 |
| USA | 2002 | 3977 |
