# Supplementary material for: Decomposing cross-country differences in quality adjusted life expectancy: the impact of value sets
Source: Popul Health Metr. 2011 Jun 23;9:17. doi: 10.1186/1478-7954-9-17 (PMC3146826; doi:10.1186/1478-7954-9-17)

**Observed and predicted HRQoL by country, gender and age (UK value set)**

**QALE using observed HRQoL vs QALE using predicted HRQoL (at age 20 for all country-gender strata and using UK values)**


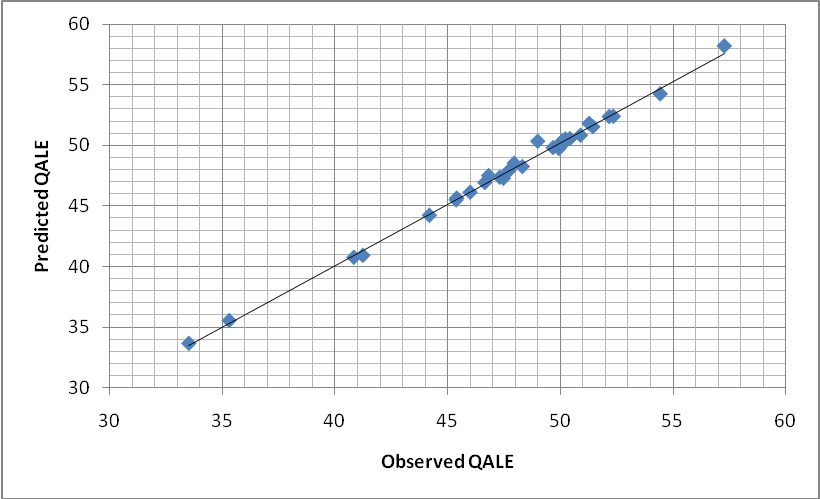

Supplement: Additional file 2 — Observed and predicted HRQoL and QALE by country, gender and age (UK value set). The figures show the observed HRQoL by country, gender and age group. Additionally, the HRQoL by country, gender and age group as predicted by the regression model is shown (the line in each figure). The last figure demonstrates QALE at age 20 using the observed HRQoL vs. QALE at age 20 using the predicted HRQoL. [file 1478-7954-9-17-S2.DOC]
