# Supplementary material for: Decomposing cross-country differences in quality adjusted life expectancy: the impact of value sets
Source: Popul Health Metr. 2011 Jun 23;9:17. doi: 10.1186/1478-7954-9-17 (PMC3146826; doi:10.1186/1478-7954-9-17)

**HRQoL score associated with different EQ-5D profiles according to six value sets**

To clarify: each point on the x-axis represents a hypothetical answer on the five EQ-5D domains: mobility, self-care, usual activities, pain/discomfort and anxiety/depression. Each domain contains 3 levels: no problems (1), some problems (2), and extreme problems (3).


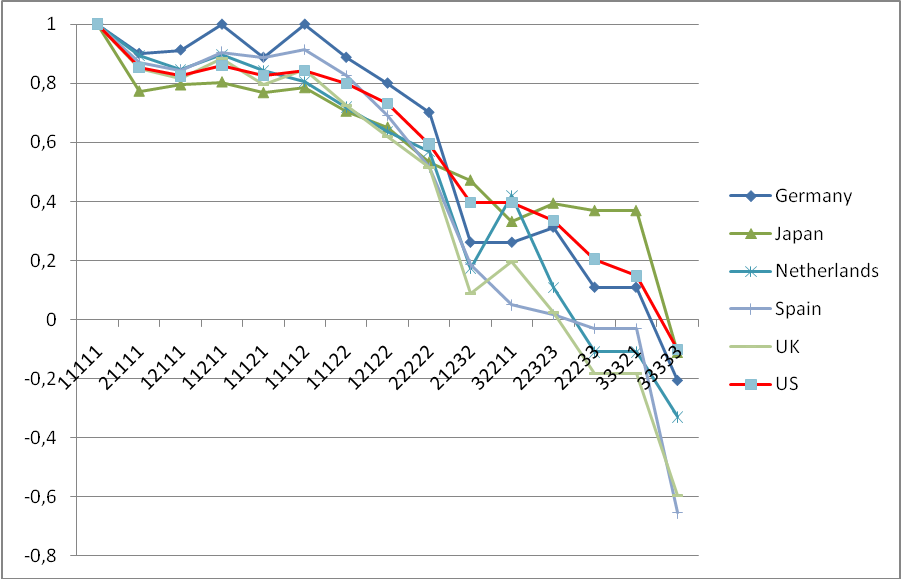

Supplement: Additional file 3 — HRQoL score associated with different EQ-5D profiles according to six value sets. HRQoL score associated with different EQ-5D profiles according to the six value sets. Each point on the x-axis represents a hypothetical set of answers in the five EQ-5D domains: mobility, self-care, usual activities, pain/discomfort and anxiety/depression. Each domain contains 3 levels: no problems (1), some problems (2), and extreme problems (3). [file 1478-7954-9-17-S3.DOC]
